# Geographical Distribution of Colorectal Cancer in Southwestern Iran Between Years 2011 and 2019

**DOI:** 10.5152/tjg.2023.22666

**Published:** 2023-10-01

**Authors:** Changiz Rostami, Arghavan Feyzmanesh, Arezoo Karimi, Salman Daliri

**Affiliations:** 1 Cancer, Environmental and Petroleum Pollutants Research Center, Ahvaz Jundishapur University of Medical Sciences, Ahvaz, Iran; 2 Bahar Hospital, Shahroud University of Medical Sciences, Shahroud, Iran; 3 Department of Epidemiology, Shahroud University of Medical Sciences Faculty of Public Health, Shahroud, Iran; 4 Clinical Research Development Unit, Imam Hossein Hospital, Shahroud University of Medical Sciences, Shahroud, Iran

**Keywords:** Geographical distribution, colorectal cancer, cumulative incidence, Iran

## Abstract

**Background/Aims:**

Colorectal cancer is one of the most common cancers in the world. Various genetic, individual, and environmental factors are associated with this disease. Today, the role of environmental and geographical factors has been considered. Accordingly, the present study was conducted to determine the cumulative incidence and geographical distribution of colorectal cancer in Khuzestan province.

**Materials and Methods:**

This study was performed ecologically to determine the cumulative incidence of colorectal cancer and its geographical distribution in Khuzestan province between 2011 and 2019. The required information was extracted from the cancer registration program of the Ministry of Health and after refinement and replication.

**Results:**

The cumulative incidence of colorectal cancer in Khuzestan province between 2011 and 2019 was estimated at 40.18 per 100 000 people. The highest cumulative incidence was related to Ahvaz city (95.87 per 100 000 people) and the lowest was related to Dasht-Azadegan city (25.35 per 100 000 people). The highest cumulative incidence of colorectal cancer, based on climate conditions, is in dry geographical areas (63.77 per 100 000 people), based on average annual rainfall, is in areas with an average rainfall of 100-200 mm (66.28 per 100 000 people), and based on land use, belonged to areas with agricultural use (74.57 per 100 000 people).

**Conclusion:**

The cumulative incidence of colorectal cancer is relatively high in Khuzestan province. The cumulative incidence of the disease was higher in the central regions of the province than in other regions. Also, based on geographical areas, the cumulative incidence of the disease was higher in areas with arid climates, the average rainfall was 100-200 mm, and in areas with agricultural land use.

**Main Points**

- The cumulative incidence of colorectal cancer in the center of Khuzestan province is higher than in other regions.
- The cumulative incidence of colorectal cancer was higher in areas with dry weather conditions and average annual rainfall of 100-200 mm.
- The cumulative incidence of colorectal cancer was higher in areas with agricultural use.
- Geographical conditions are related to the occurrence of colorectal cancer.

## Introduction

Cancers are one of the most important public health issues and the second cause of morbidity and mortality in the world. One of these common cancers is colorectal cancer (CRC). Among all cancers, CRC ranks second in mortality (9.2%) and third in diagnosis (6.1%).^1^ Colorectal cancer involved 1.8 million people as new cases and lead to the death of 881 000 patients in 2018.^2^ According to the Global Organization Board of Cancer Association Network (GLOBOCAN), there were 50 000 new patients with anal cancer and 0.7 million and 1.5 million new patients with rectal and colon cancer, respectively. It is estimated that the total number of new cases will more than double in 2040.^2^ In 2020, CRC caused 419 536 deaths among women and 515 637 deaths among men in the world.^3^ In addition, the incidence of CRC in men was 30% higher than in women.^4^ The highest prevalence of CRC in the world in 2018 belonged to Asia (51.8%) and the lowest prevalence in Oceania (1.2%).^5^ According to a 2012 study, North America, New Zealand, Europe, and Australia had the highest incidence of CRC, and Central America, South Central Asia, and Africa had the lowest incidence of CRC.^6^ According to the World Health Organization in 2020, Southern Europe (25.3%), Northern Europe (23.2%), New Zealand (22.8%), and Hungary (21.1%) are the highest new cases of CRC. Eastern Europe (16.9%) and Northern Europe (15.1%) had the highest number of new cases of rectal cancer.^7^ Iran is located in the region of Southwest Asia. According to a study conducted in 2020, the prevalence of CRC among Iranian men and women was 11.6-6.9 and 10.5-6.3 per 100 000 people, respectively. Colorectal cancer was also the fourth leading cause of cancer death in women (8.4%) and the fifth leading cause of death in men (7.5%).^8^ According to the results of the study, the northern, central, and western provinces of Iran had the highest prevalence and the southeastern provinces had the lowest prevalence of CRC.^9^

Various risk factors for CRC have been considered. In recent years, with increasing economic development and civilization, as a result of low physical activity and overweight, CRC has increased in many countries.^10^ Other risk factors include genetic factors, environmental factors, gender, schistosomiasis, smoking, alcohol, excessive consumption of red meat especially processed meat, low consumption of fruits and vegetables, and a Western lifestyle.^11^ Obesity, alcoholism, physical activity, and genetics are the most common risk factors in high-income countries.^12^ One of the factors that play an important role in the distribution of CRC in the world is the different geographical conditions. According to studies conducted around the world, the incidence and prevalence of this cancer have varied in different geographical areas.^13^ In 2018, the highest incidence of CRC in the world was in Europe, Eastern Europe, East Asia, and North America, and the lowest in Africa and Southeast Asia.^14^ In the study in Asia, the highest incidence of CRC was in China, Japan, Korea, and Turkey, and lowest incidence in India, Pakistan, Afghanistan, and Iraq.^15^ In Iran, the highest incidence of CRC belonged to the provinces of East Azerbaijan, Gilan, Tehran, Mazandaran, and Isfahan and the lowest incidence belonged to the provinces of Kerman, Sistan, and Baluchestan, and Hormozgan, which have different geographical conditions.^9^ Urbanization increased CRC in Iran; it causes increased smoking, weight gain, and tobacco use. Based on the conducted study, there is a linear relationship between increasing urbanization and CRC.^16^ Temperature, rainfall, and humidity levels appear to play an indirect role in increasing cancer incidence. They are likely to increase the production of carcinogens naturally.^17^ Socioeconomic status is a notable role in the different prevalence between provinces of Iran. In the central and western provinces of Iran, the CRC industrial regions are more than other regions. Air pollution and carcinogens show an increased prevalence of CRC.^18^

In general, genetic, demographic, geographical, and environmental risk factors affect the incidence of colon cancer. Accordingly, this study was conducted to determine the incidence and geographical distribution of CRC in Khuzestan province during the years 2011 to 2019.

## Materials and Methods

This ecological study was conducted to investigate the geographical distribution of CRC and estimate the cumulative incidence in Khuzestan province, between the years 2011 and 2019. Khuzestan province is located in the southwest of Iran and has 2 mountainous and plain areas. The study population included all patients with colon and rectal cancer registered in the cancer registration system of Khuzestan province between the mentioned years. The required information was collected from the National Cancer Registration System, Disease Management Center of the Ministry of Health, Treatment, and Medical Education by Pars30 software. In this registration program, cancer registration is population based and information about clinically and pathology diagnosed primary cancers and reported tumors is collected from hospitals, screening, diagnostic, treatment, and health centers and reported to the Cancer Registration Center in the Deputy Minister of Health. The collected information is then sent to the Disease Management Center of the Ministry of Health. In this center, after being evaluated for coding, demographic information, and elimination of duplicates, the corrected information is reported to the university health deputy. This information included the demographic information, year of diagnosis, place of diagnosis, and exact location residence of the patient. This study was approved by Shahroud University of Medical Sciences (Ethical code: IR.SHMU.REC.1399.082).

### Inclusion and Exclusion Criteria

Inclusion criteria include a definitive diagnosis of CRC based on clinical and pathological criteria by relevant specialists and residents in Khuzestan province and exclusion criteria include a non-definitive diagnosis of CRC, residence in other provinces, and disease in years before the study.

For geographical mapping, including a map of Khuzestan province by separate city, average annual rainfall, vegetation, climatic conditions, and land use, required information from Housing and Urban Development Organization, meteorology and natural resources, and watershed office and by using soft ArcGIS ver. 10.3 maps were prepared. This software is useful in identifying, distributing, and investigating the geographical and temporal patterns of diseases. After entering the information about the geographical area and the occurrence of diseases in that area, this software can be used to prepare maps based on the cumulative incidence of diseases. After entering the information about the geographical area and the occurrence of diseases in that area, this software can be used to prepare maps based on the cumulative incidence of diseases in the form of images.^19^

### Statistical Analysis

To estimate the cumulative incidence in the population studied was calculated at the beginning of 2011, then, the number of patients in each area in the numerator and the denominator was the resident population in each region and by a coefficient of a hundred thousand people in the regions studied was calculated cumulative incidence. To determine the relationship between the cumulative incidence of CRC in different sectors and environmental factors, including climatic conditions and rain, the chart scatter plot and Poisson regression test were used. Statistical analysis was performed using the software Statistical Package for the Social Sciences version 22.0 (IBM Corp.; Armonk, NY, USA). Maps of climatic factors including state map, climate, land use, vegetation, and average annual rainfall were performed using the software ArcGIS ver. 10.3. Google Earth Pro 7.3.3.7786 software was used to determine the patient’s location on the map. 

## Results

**Figure 1.**
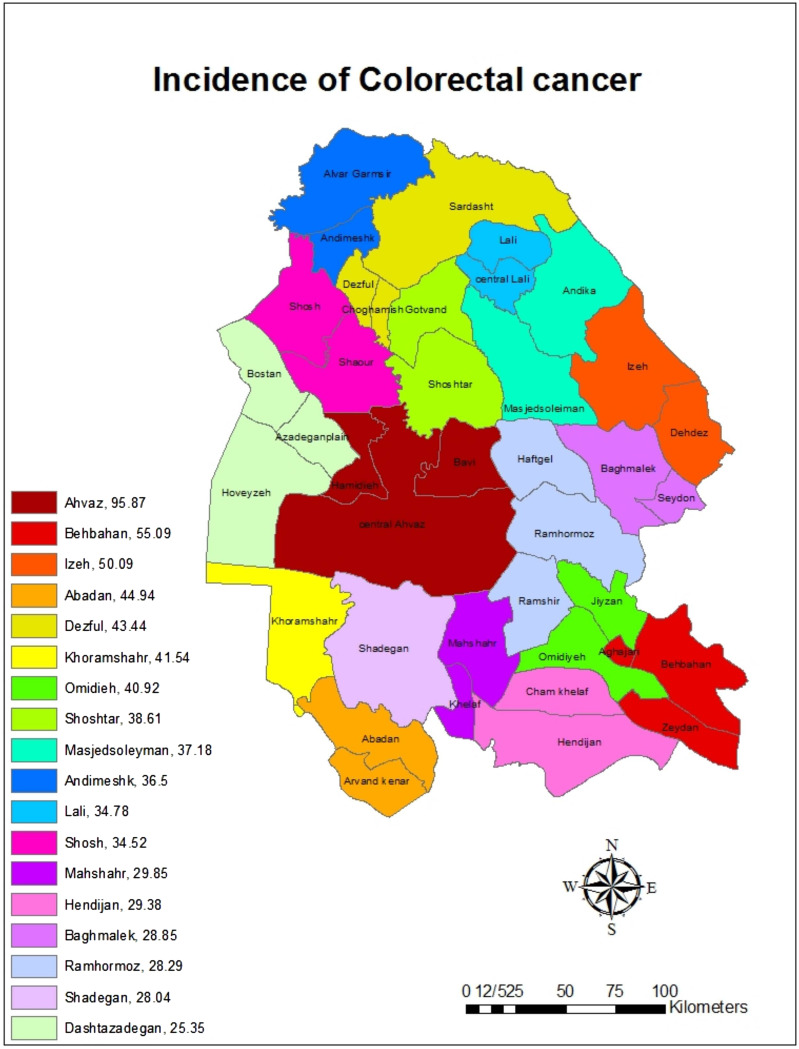
The cumulative incidence (per 100 000 people) of colorectal cancer in the cities of Khuzestan province by using GIS during the years from 2011 to 2019.

A total of 1821 patients with CRC registered in Khuzestan province between 2011 and 2019 were studied. Based on the findings, the cumulative incidence of CRC during the mentioned years in Khuzestan province was estimated at 40.18 per 100 000 people. Among the cities of Khuzestan province, the highest cumulative incidence belonged to Ahvaz (95.87 per 100 000 people) and Behbahan (55.09 per 100 000 people) and the lowest cumulative incidence belonged to Dasht Azadegan (25.35 per 100 000 people) and Shadegan (28.04 per 100 000 people), respectively (Figure 1).

**Figure 2.**
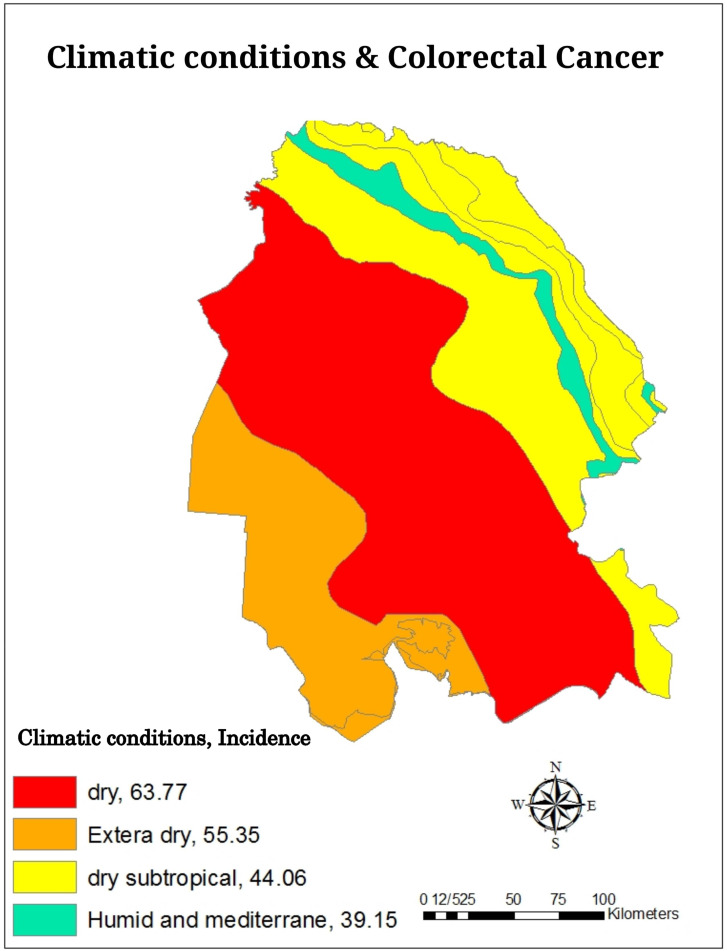
The cumulative incidence (per 100 000 people) of colorectal cancer based on climatic conditions in Khuzestan province by using GIS during the years from 2011 to 2019.

**Figure 3.**
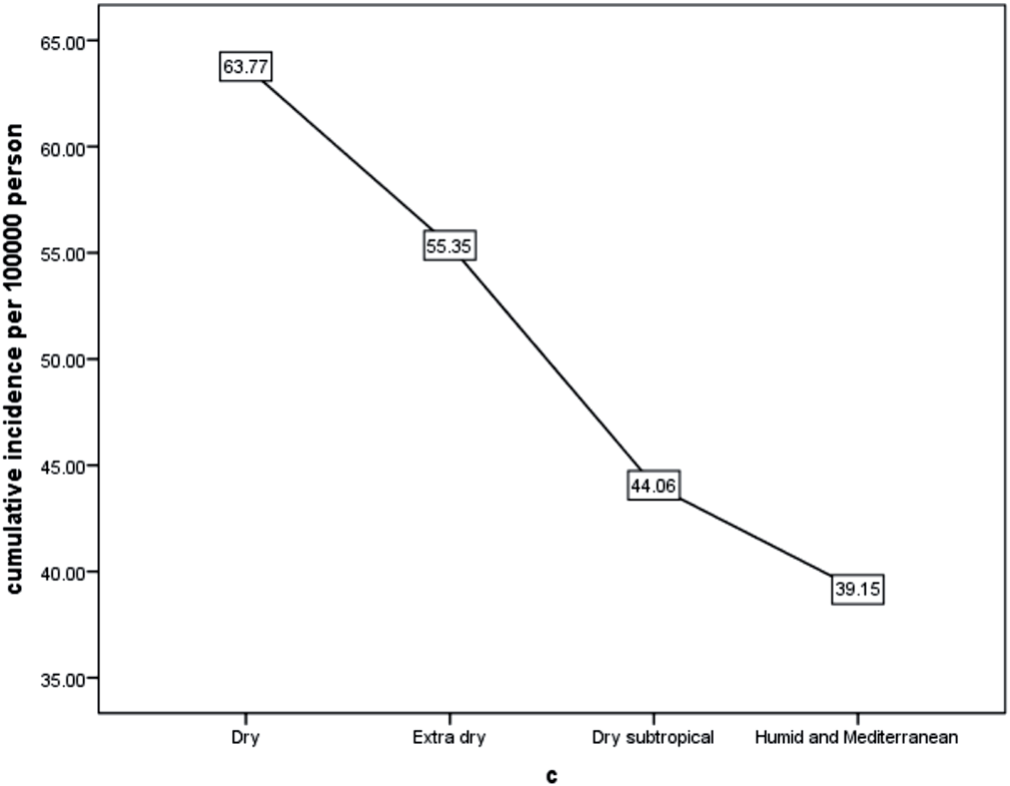
The relationship between the cumulative incidence of colorectal cancer and climatic conditions in Khuzestan province during the years from 2011 to 2019.

**Figure 4.**
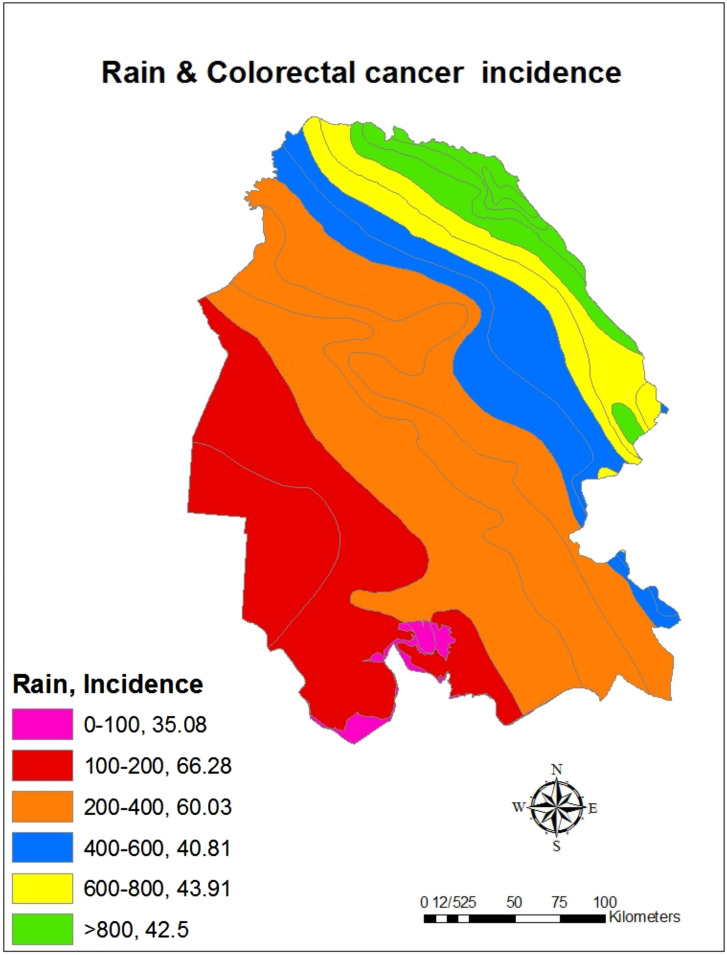
The cumulative incidence (per 100 000 people) of colorectal cancer based on average annual rainfall (mm) in Khuzestan province by using GIS during the years from 2011 to 2019.

**Figure 5.**
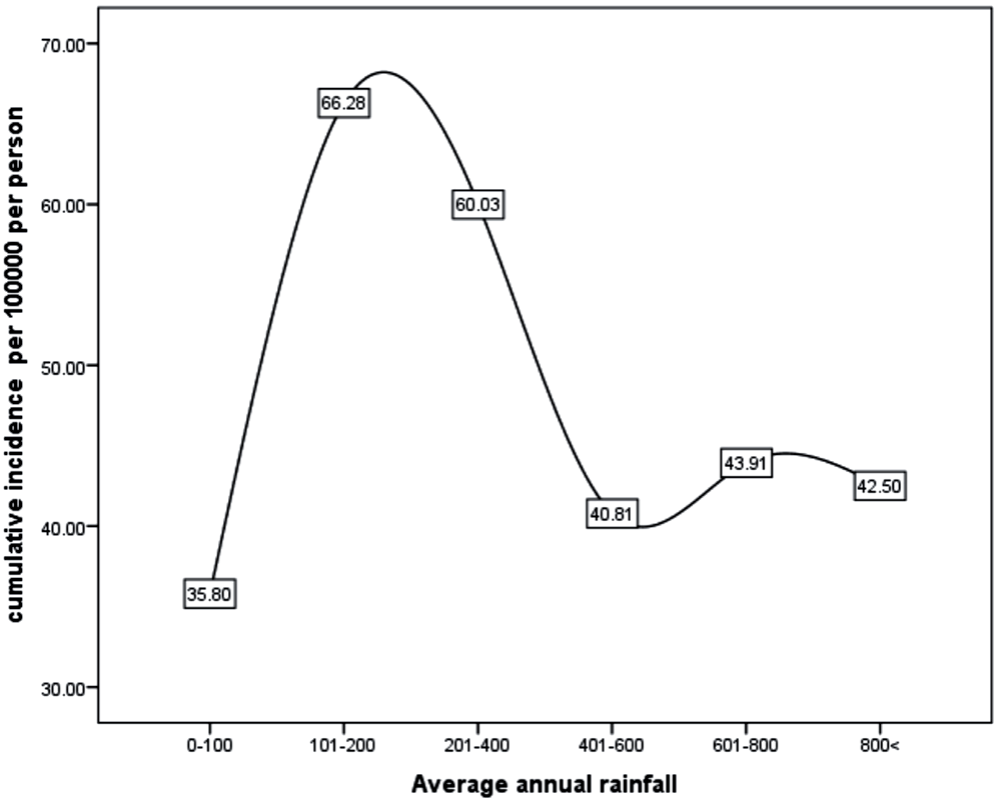
The relationship between the cumulative incidence of colorectal cancer and average annual rainfall in Khuzestan province during the years from 2011 to 2019.

The investigation of cumulative incidence of CRC based on climatic conditions showed that the highest cumulative incidence belonged to dry climates (63.77 per 100 000 people) and the lowest cumulative incidence belonged to humid and Mediterranean climates (39.15 per 100 000 people) (Figures 2 and 3). Also, the investigation of the cumulative incidence of the disease based on the average annual rainfall showed, although in areas with an average rainfall of less than 100 mm, the cumulative incidence of the disease was low, in areas with an average rainfall of 100-400 mm more cumulative incidence and areas with average annual rainfall more than 400 mm, the cumulative incidence of the disease was reduced (Figures 4 and 5).

**Figure 6.**
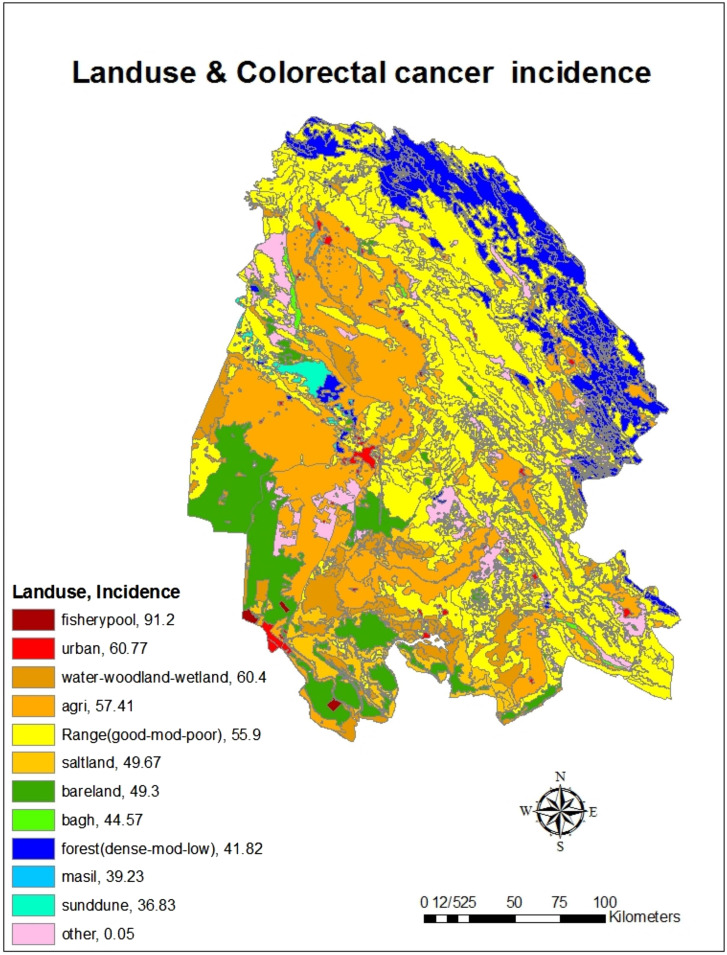
The cumulative incidence (per 100 000 people) of colorectal cancer based on land use in Khuzestan province by using GIS during the years from 2011 to 2019.

**Figure 7.**
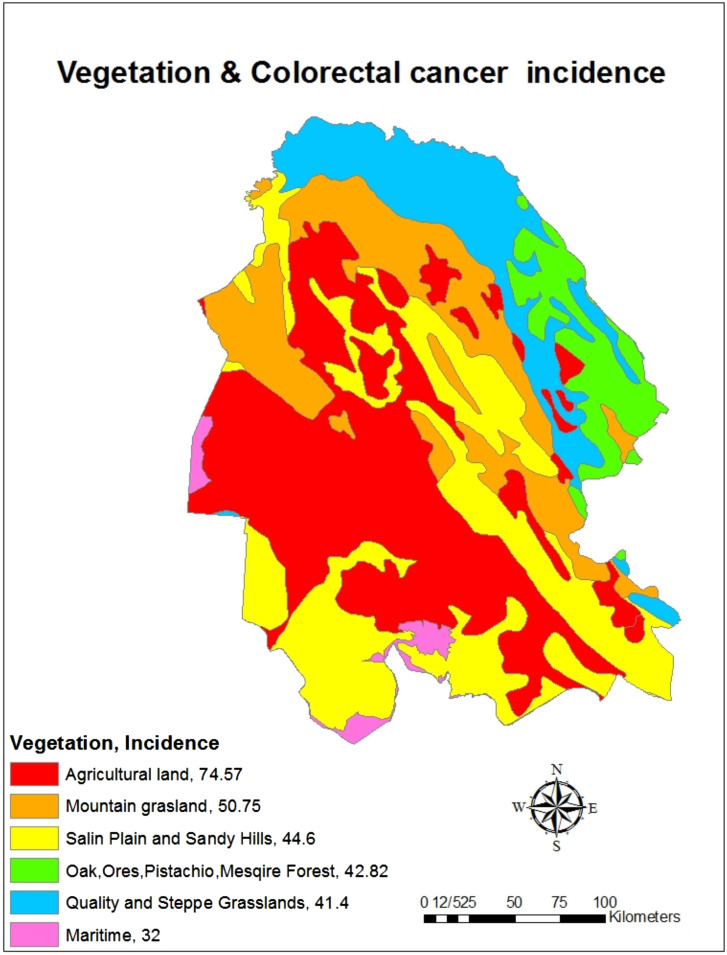
The cumulative incidence (per 100 000 people) of colorectal cancer based on vegetation in Khuzestan province by using GIS during the years from 2011 to 2019.

Estimation of cumulative incidence of CRC based on land use showed that the highest cumulative incidence of the disease was in fishery pools (91.2 per 100 000 population) and urban (60.77 per 100 000 population) and the lowest cumulative incidence was in sand dunes (36.83 per 100 000 population) and masils (waterway) (39.23 per 100 000 people) (Figure 6). Also, in the field of cumulative incidence of the disease based on vegetation, the highest cumulative incidence belonged to agricultural areas (74.57 per 100 000 people) and the lowest cumulative incidence belonged to coastal areas (32.0 per 100 000 people) (Figure 7).

## Discussion

According to the findings in this study, the cumulative incidence of CRC in Khuzestan province is estimated at 40.18 per 100 000 people. In the study by Dehghani et al,^20^ the incidence of CRC considerably raises from 6.48 in 2003 to 9.34 patients per 100 000 persons in 2010 in Iran. In the study by Enayatrad et al,^21^ it was reported that the highest incidence of CRC among females was estimated in Semnan (80.14% per 100 000 people), Tehran (47.14 per 100 000 people), and Eastern Azerbaijan (82.12 per 100 000 people) and among men it was estimated in Eastern Azerbaijan (41.14 per 100 000 people), Semnan (62.13 per 100 000 people), and Tehran (78.16 per 100 000 people). Wong et al^15^ studied the prevalence of CRC in Asia. Accordingly, the prevalence of CRC in Iran was 24.8-34.5 per 100 000 people. But among Iran’s neighboring countries, the prevalence in Turkey is <46.5; in Saudi Arabia, Bahrain, Qatar, and the United Arab Emirates between 34.5 and 5.5; in Oman 24.5-8.5; and in Iraq <13.5. It was reported in 100 000 people. This shows that the prevalence of this cancer is higher in some neighboring countries of Iran and lower in some.^15^ A study by Shadmani et al^9^ investigate the incidence of CRC in Iran based on data from the 2008 Cancer Registration Program: East Azarbaijan, Guilan, Mazandaran, Tehran, and Isfahan provinces had high incidence; Khuzestan, Fars, Yazd, and Kermanshah provinces had mild incidence; and Bushehr, Kohgiluyeh and Boyer-Ahmad, Chaharmahal Bakhtiari, Lorestan, and Ilam provinces had a low incidence of CRC. In general, Iran is one of the regions with a moderate incidence of CRC in the Middle East. Khuzestan province in the southwestern region of Iran has an average incidence among the provinces of the country. But the incidence is relatively higher compared to Iraq, which is neighboring this province and neighboring provinces.

In our study, the highest cumulative incidence of CRC and the lowest were seen in dry climate and humid areas, respectively. Also, in terms of average annual rainfall, the highest cumulative incidence belonged to areas with 100-400 mm and the lowest cumulative incidence belonged to areas with less than 100 mm. Also, in areas with more than 400 mm of rainfall, the incidence has decreased again. Li et al^22^ showed that areas with high forest coverage and humidity were beneficial for the immune system and caused decreased incidence of CRC. According to the study by Aminzadeh et al,^23^ the Eastern parts of Golestan province and the Turkmen Sahra with dry weather had the highest incidence of cancers. Shah et al^17^ found that CRC incidence in very cold climates and precipitation was higher than in warm and dry climates. In addition, Wu et al^24^ clarified that increased rainfall in industrial areas leads to the conversion of pollutants into carcinogens such as Nitrous acid (HONO). HONO is a harmful product for genes and converts to nitrites in groundwater, and as a result chemical transformations cause CRC.^24^ In a study by Turner et al^25^, air pollution was studied with cancer deaths. Exposure to NO_2_ increased mortality in patients with CRC. López-Abente et al^26^ investigated the association of exposure to industrial contaminants with CRC. According to the findings, proximity to the mining industry, the production of paper and wood, food and beverages, and the production and processing of metal and ceramics increased the risk of CRC.^26^ In general, exposure to air pollution and environmental pollution increases the risk of cancer. In general, exposure to air pollution and oil and environmental pollution increases the risk of cancer. One of the factors that can reduce these pollutants is rainfall. Increasing the intensity of rainfall will reduce this pollution. Khuzestan province is exposed to pollution caused by fine dust annually and also due to having oil and gas reservoirs. In the arid regions of the province, due to the decrease in rainfall, these pollutants remain in the environment for a longer period and increase the exposure to it, but in the wet areas, the amount of pollution decreases due to the increase in precipitation. But how much rainfall is needed to eliminate these contaminants needs further investigation. In our study, the incidence of CRC increased from 100 to 400 mm precipitations but then decreased with increasing precipitation. This is because heavier rainfall may reduce exposure to pollution. Of course, it should be noted that pollution and rainfall alone are not responsible for the disease, and various factors such as race, genetics, and lifestyle are not interactively affecting the disease.

In this study, the highest cumulative incidence belonged to agricultural areas and the lowest cumulative incidence belonged to coastal areas. A study by Matich et al^27^ demonstrates the relationship between CRC and contact with pesticides. Varghese et al^28^ revealed that pesticides including organochlorine and organophosphate increased CRC risk among people who work and live in agricultural areas, significantly. In a study by Chen et al^29^ in China, an increase in organochlorine toxins including hexachlorocyclohexane (HCH) and dichlorodiphenyltrichloroethane (DDT) in rice and soil increased the risk of CRC. In a study by Purdue et al^30^ in the United States, occupational exposure to organochlorine toxins such as chlordane and aldrin increased the risk of CRC. Abolhassani et al^31^ investigated the relationship between exposure to organochlorine and organophosphate toxins and CRC as a case–control study. The results showed that the mean serum levels of organochlorine toxins (α-HCH, β-HCH, γ-HCH, 2,4-DDE, 4,4-DDE, 2,4-DDT, and 4,4-DDT) in patients were significantly higher than in healthy individuals. Also, the mean serum levels of malondialdehyde and total antioxidant capacity in the serum of the patient group were significantly higher than the control group.^31^ Xu et al^32^ suggested that the consumption of fresh fish in the diet can reduce the risk of CRC in the coastal area by 53%. In a review study by Shivappa et al^33^, the Dietary Inflammatory Index (DII) was associated with CRC, with increasing or decreasing CRC increasing CRC and decreasing DII. Anti-inflammatory food ingredients include unsaturated fatty acids, omega 3, omega 6, fiber, niacin, riboflavin, thiamine, magnesium, zinc, selenium, vitamin A, vitamin B6, B12, vitamin D, vitamin C, vitamin E, beta-carotene, and folic acid. Pre-inflammatory dietary components included total fat, trans fat, cholesterol, saturated fatty acids, proteins, carbohydrates, vitamin B12, and iron.^33^ In general, habitat causes a change in exposure to environmental factors affecting CRC, including exposure to environmental pollutants and poisons that may be man-made or naturally occurring, such as air pollution or spraying to repel plant pests. It is also another factor influencing the type of nutrition because people’s nutrition is related to the availability of food. As a result, vegetables and red meat are used more in agricultural areas, but fish and seafood are used more in coastal areas, which can be effective in causing this cancer.

Limitations of this study included (1) cumulative incidence based on geographical conditions was investigated ecologically, which can cause ecological fallacy; (2) uncertainty of individual characteristics of the studied patients such as race, social status, and lifestyle; (3) intra-provincial and extra-provincial migration of some patients; and (4) the possibility of underreporting due to not identifying all patients.

## Conclusion

The cumulative incidence of CRC is relatively high in Khuzestan province. The cumulative incidence of the disease was higher in the central, southern, and southwestern regions of the province than in other regions. Also, based on climatic conditions, the cumulative incidence of the disease was higher in areas with arid climates; the average rainfall was 100-200 mm and in areas with agricultural land use. It is suggested that individual-level studies be performed to determine the role of these factors in CRC.
